# Instruction in Microscopy

**Published:** 1852-02

**Authors:** 


### INSTRUCTION IN MICROSCOPY.

Our readers will find in the advertising sheet, the announcement of a course of lectures and demonstrations on the above subject, by Dr. John Neill, Demonstrator of Anatomy in the University of Pennsylvania.

To those desirous of pursuing this most interesting department of Medical Science, we can confidently recommend Dr. Neill as in every respect competent to teach it. It will be seen that the number of the class is limited to twelve.

